# Senolytics: A Novel Strategy for Neuroprotection in ALS?

**DOI:** 10.3390/ijms222112078

**Published:** 2021-11-08

**Authors:** Alexandra Maximova, Eryn L. Werry, Michael Kassiou

**Affiliations:** 1 Faculty of Medicine and Health, The University of Sydney, Sydney, NSW 2008, Australia; amax8835@uni.sydney.edu.au; 2 Faculty of Science, The University of Sydney, Sydney, NSW 2008, Australia

**Keywords:** amyotrophic lateral sclerosis, senescence, senolytics, neurodegenerative disease

## Abstract

Amyotrophic lateral sclerosis (ALS) is a progressive motor neurodegenerative disease that currently has no cure and has few effective treatments. On a cellular level, ALS manifests through significant changes in the proper function of astrocytes, microglia, motor neurons, and other central nervous system (CNS) cells, leading to excess neuroinflammation and neurodegeneration. Damage to the upper and lower motor neurons results in neural and muscular dysfunction, leading to death most often due to respiratory paralysis. A new therapeutic strategy is targeting glial cells affected by senescence, which contribute to motor neuron degeneration. Whilst this new therapeutic approach holds much promise, it is yet to be trialled in ALS-relevant preclinical models and needs to be designed carefully to ensure selectivity. This review summarizes the pathways involved in ALS-related senescence, as well as known senolytic agents and their mechanisms of action, all of which may inform strategies for ALS-focused drug discovery efforts.

## 1. Amyotrophic Lateral Sclerosis: Prevalence, Causes, and Symptoms

Amyotrophic lateral sclerosis (ALS) is a devastating degenerative disease with a 3 in 100,000 prevalence in Europe [1]. The incidence of ALS displays a positive correlation with increasing age and the average age of onset is 55–65 years old [2]. The average ALS survival period is 3–5 years post-diagnosis [3], with disease end stage often resulting from progressive paralysis leading to respiratory failure [4]. Motor neuron deterioration usually begins at a random location within the upper or lower motor neurons before spreading to other regions of the motor cortex and spinal cord [5]. Due to the variation in the anatomical origin of the disease, the symptom presentation differs greatly between individuals [5]. While lower motor neuron degeneration results in muscle weakness and fasciculations, damage associated with upper motor neurons can present through hyperreflexia and high muscle tone [4]. Furthermore, ALS can manifest in bulbar symptomology in approximately a quarter of affected individuals, resulting in an inability to speak or swallow due to the paralysis of associated muscles required for voluntary control [4]. Several techniques are used in ALS diagnosis. These include electromyography to measure electrical activity in muscle and nerve conduction studies to measure speed of conduction in neurons [2]. Blood and urine samples can provide information about elevated signature inflammatory markers, while MRI scans inform about the individual’s cerebral degeneration on a gross scale [2].

The exact cause of ALS is unknown, with familial inheritance (fALS) only contributing to ~10% of all cases [6], while the remaining 90% are sporadic (sALS). The two most common mutations associated with ALS are in chromosome 9 open reading frame 72 (C9orf72) and superoxide dismutase (SOD1). The *C9orf72* gene is important for autophagy and vesicle transport across membranes [2] and various mutations in this gene contribute to almost half of fALS cases and 7% of sALS [4]. In healthy individuals, there are no more than 30 GGGGCC repeats in this gene [7]. People who develop ALS from a C9orf72 mutation show hundreds or thousands of repeats [5]. Mutations in SOD1 make up 20% of fALS cases [3] and 1% of sALS cases [5]. In healthy individuals, the SOD1 enzyme plays an important role in antioxidant defence by metabolising reactive oxygen species (ROS) into hydrogen peroxide and oxygen [7]. Amongst other pathomechanisms, genetic mutations associated with this enzyme result in protein misfolding [7] and toxic aggregate accumulation in the mitochondria [8]. The mutated aggregates reduce protection against ROS due to loss of function and affect the mitochondria’s ability to balance the calcium gradient [8], contributing to inflammation and neurodegeneration.

Currently, there is no effective disease-altering pharmacological or alternative intervention available for ALS, with very few FDA-approved pharmaceutical agents. These currently approved drugs include glutamate release inhibitor riluzole, an N-methyl-D-aspartate (NMDA) receptor antagonist Nuedexta, used to treat some of the symptoms associated with the bulbar manifestation of ALS [9], and edaravone, which targets free radicals [10]. However, while these agents have shown some efficacy in managing symptoms and slowing disease progression, they do not provide a cure and only extend the average survival time by mere months. This highlights the need for identifying new therapeutic approaches that could lead to a disease-modifying therapy.

## 2. Cellular Senescence in ALS

A new therapeutic approach with potential to treat ALS may be through targeting cellular senescence. Senescence is a non-replicative state that cells enter upon repeated stress and injury related to recurring DNA damage and telomere shortening [11], thought to have evolved as a protective mechanism against tumour generation. Senescent cells possess a unique morphology with large, flat cell bodies [12] and an altered nuclear envelope [13]. Once senescent, cells show resistance to apoptosis and display dysregulation of molecules involved in the cell cycle and proliferation. They release a range of pro-inflammatory molecules, which impart stress on surrounding cells. Senescent cells also lose their normal functionality, removing their regular supporting role and placing further stress on surrounding cells. Although there is no universal marker of senescence, the presence of a combination of these indicators is often used to identify senescence, and there is mounting evidence that these hallmarks are present in ALS.

### 2.1. Senescence-Associated Beta-Galactosidase

One established marker of senescence is the expression of senescence-associated beta-galactosidase (SA-β-Gal) which is significantly higher in senescent cells, differentiating them from their normally-proliferating or quiescent counterparts [14]. Higher SA-β-Gal levels are found in the spinal cords of SOD1^G93A^ overexpressing rats [15,16]. Astrocytes cultured from these spinal cords also show significantly higher SA-β-Gal expression and decreased cell death compared to age-matched wild-type cells [15], suggesting the involvement of these pathways in ALS. Similarly, spinal cord astrocytes derived from induced pluripotent stem cells (iPSCs) of ALS patients with the C9orf72 mutation displayed a significant increase in SA-β-Gal expression and a reduction in the proliferation-related marker Ki-67 overtime, supporting the involvement of senescence in the disease and its age-dependent development [17].

### 2.2. Apoptosis-Related Pathways

**Figure 1:**
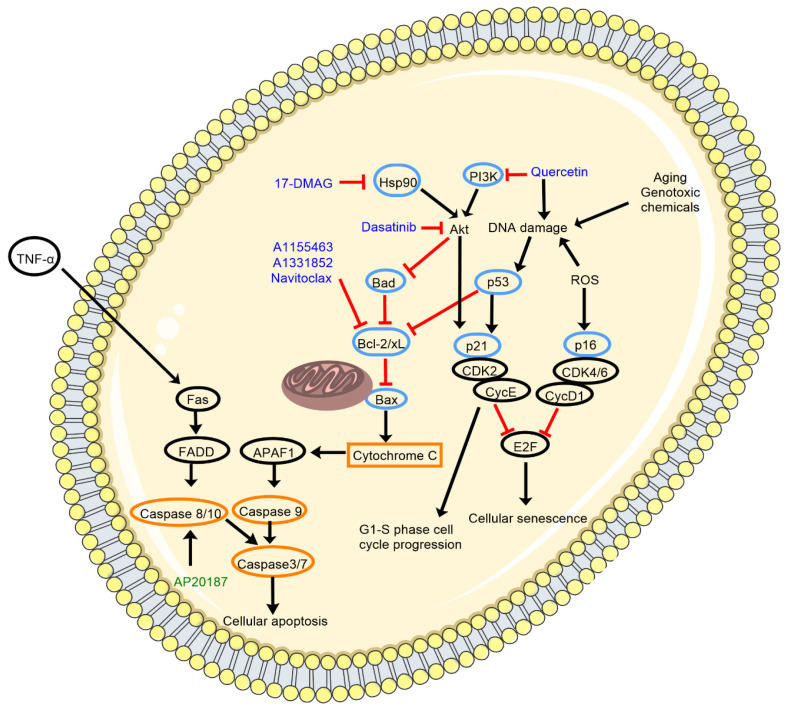
Simplified schematic of signaling pathways involved in senescence and senolysis. Stimuli such as aging, genotoxic chemicals and reactive oxygen species (ROS) activate key targets such as p53, p21, and p16. This results in senescence via interactions with cyclins and cyclin-dependent kinases, and resistance to apoptosis via inhibition of the Bcl-2/Bcl-xL pathway. Important protein targets and apoptotic signaling molecules are identified with blue and orange outlines, respectively. Senolytic drugs are annotated in blue text. A tool to trigger senolysis in the INK-ATTAC model is noted in green text. Black arrows represent activation. Arrows do not always indicate direct activation. Red blunted-ended lines represent inhibition. Hsp90: heat shock protein 90; PI3K: phosphoinositide 3-kinase; Bad: Bcl-2-associated death promoter; Bcl-2: B-cell lymphoma 2; Bcl-xL: B-cell lymphoma extra-large; ROS: reactive oxygen species; CDK: cyclin-dependent kinase; Cyc: cyclin; APAF1: apoptotic protease activating factor 1; TNF- α: tumour necrosis factor α; FADD: Fas-associated death domain.

To evade cellular death, senescent cells upregulate anti-apoptotic genes [18] whilst simultaneously downregulating proapoptotic mechanisms. One of these pathways involves the B-cell lymphoma 2 (Bcl-2) protein family members, including Bcl-2 and B-cell lymphoma extra-large (Bcl-xL). Overexpression of these proteins prevents cellular death in response to exposure to damaging stimuli such as oxidative species such as H_2_O_2_ [19]. Activation of Bcl-2 induced by DNA damage results in Bcl-2 binding to, and consequently inhibiting, the proapoptotic protein Bax, which would usually trigger downstream release of caspases that can induce cell death (Figure 1). Overexpression of Bcl-2 is also positively correlated with SA-β-Gal activity, suggesting its role in development of senescence [19]. Other molecules involved in the anti-apoptotic pathway include phosphoinositide 3-kinase (PI3K) and heat shock protein 90 (Hsp90), both of which are upregulated in senescent cells, allowing them to resist apoptosis and increase cellular survival through activating downstream Bcl-2 signaling [20,21] (Figure 1).

Hetz and colleagues [22] showed that Bcl-2 and Bcl-xL mRNA and protein levels were increased in the spinal cord of symptomatic SOD1^G85R^ mice. While Peviani et al. [23] reported no significant difference in activation of PI3K in motor neurons from the spinal cords of SOD1^G93A^ mice compared to healthy controls, they showed that PI3K-induced signaling was upregulated in astrocytes residing in the ALS-affected regions [23], revealing an association between the role of glial senescence and subsequent resistance to apoptosis in disease progression.

### 2.3. Changes in Cell Cycle Inhibitor Levels

The cyclin-dependent kinase inhibitor p16, sometimes referred to as p16INK4a, slows cell cycle progression from the G_1_ to the S-phase, contributing to senescence [24]. Upregulation of p16 is one of the most widely used markers of senescence [25]. Cells expressing p16 are increased four-fold in the lumbar spinal cord of symptomatic rats expressing the SOD1^G93A^ mutation compared to wild-type controls or non-symptomatic mutants [16]. Expression of p16 is also significantly elevated in the frontal and motor cortices of post-mortem ALS brains when compared to the same regions in age-matched healthy human tissue [26]. The cell types expressing upregulated p16 appear to depend on the state of disease progression, species, and region within the central nervous system. In SOD1^G93A^ mutant mice, lumbar spinal cord microglia were the primary cell type exhibiting increased p16 expression, which was evident at paralysis onset and continued to increase throughout progression of the disease [16]. Astrocytes and motor neurons also displayed p16 staining, but only in advanced stages of the disease [16]. In contrast, elevated p16 expression was found in astrocytes and other glia, but not neurons, in the frontal association cortex of human ALS donors [26]. However, no increase was seen in any cell type in the motor cortex of these donors [26].

Another commonly used marker to detect senescent cells is upregulation of a second cyclin-dependent kinase inhibitor, p21 [27]. This p21 plays a role in arresting the cell cycle through inhibiting the formation of the cyclin/cyclin-dependent kinase protein complex, which is crucial for cell division and proliferation in the G_1_ and S cell cycle phases [18] (Figure 1). Increased p21 expression is often associated with an overall dysregulation in cell cycle arrest and is elevated in both neurons and glia in the frontal association cortex, but not motor cortex, of ALS-affected brains [26]. In particular, p21 was shown to be upregulated in C9orf72 astrocytes derived from ALS patients [17], as well as in astrocytes cultured from SOD1^G93A^ overexpressing mutant rats [15].

A third marker commonly used to detect senescence is upregulation of the cell cycle inhibitor, p53, which regulates p21 levels (Figure 1). There was a 1.5-fold increase in p53-positive cells in the lumbar spinal cord of SOD1^G93A^-overexpressing rats when paralysis first appeared compared to wild-type controls, and continued to increase throughout disease progression [16,22]. Such p53 upregulation has been noted in motor neurons and astrocytes of the post-mortem human spinal cord and motor cortex of ALS patients [28]. Furthermore, under severe cellular stress, individuals with ALS exhibit inhibition of the enzyme MDM2 that is responsible for the degradation of p53 [29], evident through significant reduction in the protein’s metabolites [28], leading to increased p53 expression due to reduced metabolism.

It should be noted that a specific marker able to detect senescence across the entire time course of senescent development does not exist. p16 may only be upregulated in later stages of senescence, implying that it may not mark cells in the early phases of senescence induction [27]. In contrast, p21 is expressed at its highest levels when cells are developing senescence and then expression levels decline once senescence is induced [27]. Additionally, in some cell types it not only marks senescent cells but also cells that have undergone DNA damage [27,30], although this might not be the case in ALS [26]. Furthermore, in some situations, p53 can reduce senescence [31]. Despite these caveats, the upregulation of all 3 of these markers across multiple modalities, including ALS human tissue, ALS animal models, and iPSC-derived astrocytes from ALS donors, in combination with the SA-β-Gal changes in Section 2.1, provides converging evidence suggesting extensive senescence in ALS, particularly in glial cells.

### 2.4. Senescence-Associated Secretory Phenotype

Despite losing their capacity to proliferate, senescent cells remain metabolically active and through an increase in IL-1α secretions [32] upregulate *IL-6* and *IL-8* expression [33]. This leads to secretion of high levels of proinflammatory cytokines, the build-up of which results in the senescence-associated secretory phenotype (SASP). Some suggest that the reason for this is to allow the immune system to identify these cells for clearance [32], where increased IL-6 and IL-8 secretions can lead to increased immune cell recruitment. However, senescence is significantly increased with ageing and can lead to the body’s inability to clear senescent cells at a rate matching their accumulation [32], resulting in excess neuroinflammation. The SASP can create a positive feedback loop to increase further pro-inflammatory mediators from the already-senescent cells while spreading inflammation and neurodegeneration to neighboring cells [26]. This relationship is evident in ALS, where astrocytes secrete toxic soluble factors including proinflammatory signaling molecules TNF-α [34], TGF-β, IL-6, IFN-γ, and nitric oxide [35] and overexpress the astrocytic proinflammatory marker C3 in human spinal cord and motor cortex, which has been correlated with the onset of ALS-associated symptoms in the SOD1^G93A^ mouse model of ALS [36].

In addition to the increased cytokine secretions, astrocytes in the SOD1^G93A^ model are significantly more reactive to cytokine release compared to wild type glial cells [36], contributing to the positive feedback loop of inflammation in ALS. An increase in TNF-α secretions from SOD1^G93A^ astrocytes in vitro is also correlated with subsequent elevations in several downstream molecules involved in inflammation, including prostaglandin E_2_ and leukotriene B_4_, in addition to increased activation of the inducible nitric oxide synthase enzyme [37] which can trigger nitric oxide generation and increase ROS burden. Spinal cord astrocytes in SOD1^G93A^ mice significantly increase downstream pro-inflammatory effects of signaling induced by IFN-γ [38], TNF-α [39], IL-6, IL-8, and IL-15 [15].

While an increase in proinflammatory cytokine secretion is correlated with old age, this difference is further exacerbated in elderly individuals affected by inflammation-driven conditions, such as ALS. In fact, healthy elderly controls show higher levels of anti-inflammatory regulation exhibited through more marked elevation in IL-10 plasma levels and a reduced level of inflammation compared to their age-matched ALS-affected counterparts [32]. Studies in individuals with ALS have further supported the specific involvement of the SASP in disease development, as patients display significantly elevated levels of IL-6, IL-8, and nitrite in their blood serum [40] and increased IL-2, IL-10, IFN-γ, and TNF-α plasma concentrations [41] compared to healthy age-matched controls.

The mechanism by which senescence results in SASP is poorly defined. There is preliminary evidence that some of the cell cycle inhibitor-related senescence markers may be involved in the generation of SASP. For example, p53 is upstream of NF-kB, a primary regulator of cytokine expression [42]. Furthermore, knockdown of p16 alleviates SASP in oncogene-induced senescent cells and DNA damage-induced senescence [43]. However, numerous mechanisms independent of these markers have also been implicated [44]. For example, accumulation of malfunctioning mitochondria in senescent cells can lead to SASP via release of cytoplasmic chromatin fragments. Considering the breadth of components that constitute SASP, and that the nature of factors released can differ with senescence-inducing stimuli [44], it is likely that SASP develops from multiple deleterious processes that occur during senescence.

### 2.5. Loss of Glial Function

A confounding factor in neurodegeneration that results from senescence development in the CNS glial population is loss of their usual supportive functions. Inducing senescence in human astrocytes through H_2_O_2_ [45] or X-ray [33] exposure downregulates excitatory amino acid transporter (EAAT) 1 and 2 [33]. X-ray irradiation of human astrocytes also downregulates Kir4.1 potassium transporter expression [33,45]. Due to EAAT1/2 and Kir4.1 being responsible for glutamate redistribution and potassium ion regulation, respectively, their downregulation results in an elevation of glutamate in the synapse. Furthermore, aged senescent rat cortical astrocytes display reduced glutamate uptake, reduced mitochondrial activity, and increased ROS production [46]. This combination significantly reduces the neuroprotective capacity of astrocytes, contributing to increased cell death of the neighboring neurons [46]. In ALS, this is further coupled with increased glutamate release by microglia [47]. Lastly, senescent astrocytes upregulate the expression of *GRINA* [33], which encodes the glutamate ionotropic receptor NMDA type subunit associated protein 1. The NMDA receptor is located on the post-synaptic motor neuron terminal and is highly permeable to Ca^2+^ [48]. Increased Ca^2+^ flow into motor neurons leads to increased neuroexcitation [49], which may be toxic to motor neurons.

Another factor contributing to the vulnerability of motor neurons is the protein lamin B1, which usually provides significant structural support to the motor neuron nucleus but is significantly reduced in senescent cells. Co-staining for nuclear p16 expression and lamin B1 in an SOD1^G93A^ rat model of ALS showed an inverse correlation between the two proteins, evident through a marked increase in p16-positive cells within the spinal cord and the associated decrease in their lamin B1 expression [16]. This dysregulation was also co-localized with senescent microglia that expressed elevated levels of ionized calcium binding adaptor molecule 1 (Iba1), p53, p16, and SA-β-Gal; all markers of senescence [16]. These trends are positively correlated with increasing age of cell cultures and animals, once again supporting the relevance of this mechanism in ALS and age-related senescence development.

## 3. Senescence as a Therapeutic Target in ALS and Other CNS Diseases

Given hallmark signatures of senescence are present in ALS, and that senescence involves deleterious ALS-relevant outcomes such as increased production of pro-inflammatory cytokines and loss of useful cell functions, removing senescent cells or restoring senescent cells back to normal function may be a strategy for producing a disease-modifying ALS therapy. While a study exploring the impact of clearing senescent cells on ALS progress has not yet been published, this approach has been successful in animal models of other neurodegenerative disorders and in ageing. A transgenic INK-ATTAC mouse model was developed that allowed for selective apoptosis of senescent cells upon administration of a trigger drug AP20187 (Figure 1) in a mouse *MAPT^P301S^PS19* model of tauopathies such as Alzheimer’s disease (AD) and frontotemporal dementia [50]. Administration of the trigger drug to eliminate senescent microglia and astrocytes that accumulated in this model led to a reduction in gliosis, prevention of tau phosphorylation and aggregation, and a complete recovery of short-term memory loss [50]. The beneficial effect of clearing senescent cells on neuron loss, overall tissue volume loss, and tau pathology have been further confirmed in four different AD transgenic mouse models [51]. Furthermore, senescent cell clearance in a fifth model of AD reduced neuroinflammation, amyloid-β plaque level, and cognitive deficits [52].

The useful effects of senescent cell clearance have also been shown in models of other neurodegenerative diseases and age-related CNS conditions. Clearance of senescent astrocytes in a paraquat-induced Parkinson′s disease mouse model reduced dopaminergic nigral neuron loss and improved motor function [53]. Similarly, clearance of senescence cells alleviated chemotherapy-related peripheral neuropathy [54]. Clearing senescent microglia from aging mice reduced microglial activation and the level of SASP, and improved cognitive functioning [55]. In another INK-ATTAC model, administration of the trigger drug AP20187 led to reduction in age-related cataract development in eyes, and muscle retention, independent of age at the time of administration [56], supporting the therapeutic benefit of using this approach in conditions with age as a risk factor. Further improvements included reduced levels of IL-6, IL-1α and TNF-α in adipose and skeletal muscle tissues, showing promise for treating inflammation [56]. AP20187-driven clearance of p16-expressing cells also displayed anti-inflammatory effects against SASP-related cytokines in animal models of aging [56] and in an obesity-induced model of senescence where drug treatment significantly reduced the levels of IL-1α, IL-1β, and TNF-α [57].

In addition to evidence that removal of senescent cells is useful in other neurodegenerative disorders and ageing, there is a suggestion that targeting aspects of senescence-related pathology can improve ALS. Mesenchymal stem cells from ALS patients show increased senescence, and if anti-senescence genes such as *TERT* and *ANG* are upregulated, these cells show increased ability to protect against oxidative damage [58]. Knocking out the genes responsible for coding some markers of inflammation such as IL-1α, TNF-α, and C1q reduces motor impairment in rotarod test performance by SOD1^G93A^ mice, demonstrating the useful effects of treatments that may decrease inflammation [36]. In addition, targeting increased inflammation by administering cromolyn sodium to SOD1^G93A^ mice led to reduced microglial activation and delayed the onset of associated symptoms [59]. These results were further supported by an increase in spinal cord motor neuron survival and a reduction in degeneration of neuromuscular junctions involved in tibialis muscle function [59], suggesting that targeting senescent cells which exhibit the SASP could help rescue motor dysfunction in ALS.

## 4. Senolytic Agents and Their Limitations

A major strategy for targeting senescent cells is the development of senolytic agents that selectively remove these cells. Although there are no studies to date examining the impact of senolysis in ALS, the evidence in Section 3 demonstrates the useful effect of clearing senescent cells in other nervous system disorders. Furthermore, it highlights the benefits of correcting senescence-related pathology in ALS models. These two factors suggest that senolysis may be a useful therapeutic approach in the disease. However, before progress can be made towards this, a suitable senolytic agent must be found.

### Inducing Senolysis: The Challenge of Choosing a Signaling Pathway Target

Much is understood about the signaling pathways involved in the development and progression of senescence from other disease models in which senescence emerges, including atherosclerosis, diabetes [60], cancer [61], and general age-related symptomology discussed in Section 3. These pathways centre around dysregulation of the pro- and anti-apoptotic cascade introduced in Section 2.2 (Figure 1), including general upregulation of anti-apoptotic proteins such as Bcl-2 and Bcl-xL, and upstream regulators of those proteins such as Hsp-90, PI3K, and p53. Current senolytics that have been trialled in non-CNS disorders or animal models of CNS disorders (Table 1) target these proteins.

p53 acts to eliminate cells carrying damaged DNA through reducing the expression of antiapoptotic members of the Bcl-2 protein family, including Bcl-xL and Bcl-2 (Figure 1). Whilst the proapoptotic protein Bax usually resides inside the mitochondria, p53 can downregulate binding between Bcl-2 family members and Bax, leading to the latter migrating to the mitochondrial cytosol (Figure 1). This increases the permeabilization of the mitochondrial membrane, triggering the release of cytochrome C and caspases [22] (Figure 1). This mechanism is crucial in combating senescence by inducing apoptosis in affected cells. While seeking to activate upstream proteins such as p53 may seem to be a suitable strategy for induction of senolysis compared to targeting individual downstream proteins such as Bcl-2 or Bcl-xL, it also carries significant risk of unwanted effects if used as a target in neurodegenerative disorders, including ALS. Upregulation of p53 in the spinal cord can result in an increase in Bax activity causing cellular apoptosis [22] and subsequent loss of motor neurons. Furthermore, while deleting p53 in mice protects neurons from death usually caused by DNA damage or excess glutamate elevation [29], it may trigger the development of tumours [29] due to suppression of p53′s ability to repair DNA damage. This highlights the multifaceted difficulty of targeting p53 signaling pathways to combat ALS-related senescence. Due to the many roles of p53 signaling, pursuing strategies to target proteins further downstream in the signaling pathway may be a safer option and is the path senolytic agent development has taken for non-CNS diseases.

**Figure 2:**
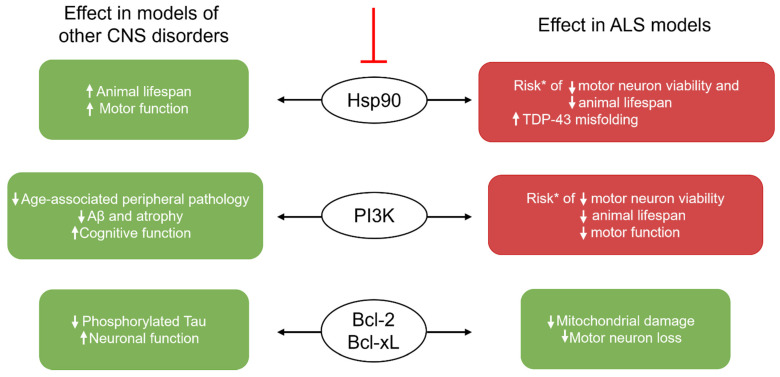
The effects of inhibiting current senolytic targets in non-ALS and ALS models. Blunted red line indicates inhibition. Red boxes and green boxes represent damaging and beneficial effects, respectively. ↑ denotes increase/improvement; ↓ denotes decrease. *Risk is based on the opposite effect occurring when Hsp90 or PI3K is activated. ALS: amyotrophic lateral sclerosis; Hsp90: heat shock protein 90; PI3K: phosphoinositide 3 kinase; Bcl-2: B-cell lymphoma 2; Bcl-xL: B-cell lymphoma extra-large.

A new class of senolytics designed to inhibit heat shock protein 90 (Hsp90) is being developed [62]. While Hsp90′s role in the development of senescence remains unclear, its role in phosphorylating Akt to its active form is well established as is its indirect upregulation of the anti-apoptotic Bcl-2 family members [20] (Figure 1). Thus, inhibition of Hsp90 prevents the binding of the protein to Akt, subsequently inducing apoptosis further downstream [63]. In this manner, the Hsp90 inhibitor 17-DMAG can prevent cells from entering cell cycle arrest [62]. This compound extends lifespan and reduces the burden of p16-positive cells in senescent embryonic mouse fibroblasts in vitro [62] (Table 1). Its senolytic efficacy was further supported in a progeroid Ercc1^−/Δ^ mouse model in vivo where it decreased the expression of p16 and SA-β-Gal and improved motor function of mice [62] (Table 1). It has also been used within the CNS, where it improved motor function and survival rate in a mouse model of polyglutamine disease [64], which offers some promise to motor neurodegenerative conditions such as ALS. Despite Hsp90 being upregulated in the serum of ALS patients [65], this approach may be hindered in ALS as Hsp90 contributes to proteostasis. Adequate proteostasis plays a key role in preventing the formation of abnormal protein aggregates that have been linked to ALS pathophysiology [66]. Pharmacological induction of Hsp90 in a SOD1^G93A^ mouse model shows efficacy in improving motor neuron viability and extending lifespan compared to untreated SOD1^G93A^ mice [15,33]. One of the proteins that Hsp90 helps to regulate proteostasis of is the trans-active DNA binding protein-43 (TDP-43), a key component of the proteinopathy in most ALS cases [67]. This is a significant consideration in CNS treatment, as deleting Hsp90 in human neuroblastoma cells leads to a significant increase in phosphorylated TDP-43 aggregates, suggesting the role of Hsp90 in the degradation of the phosphorylated aggregate [68]. These findings highlight the challenge of targeting these pathways to reduce senescence in glia whilst minimising further motor neurodegeneration (Figure 2) and represent a strong need for additional research to determine the relative extent of Hsp90 dysregulation in each of the cell types during ALS to inform future selection of an appropriate pharmacological target.

PI3K is another protein that is a current target for senolytic development. Similarly to Hsp90, activation of PI3K phosphorylates Akt, increasing its activity and resulting in upregulated Bcl-2 expression [18,69] (Figure 1). Elevated PI3K activity leads to cellular morphological changes and is correlated with increased IL-6 secretions contributing to the formation of the SASP, both of which are associated with senescence [70]. Furthermore, the involvement of the PI3K/Akt pathway in senescence is evident through its ability to upregulate expression of several markers of senescence including p53, p21, and SA-β-Gal [71], and its correlation with replicative senescence [72] in human cell lines in vitro. The senolytic agent quercetin inhibits PI3K and induces DNA damage through intercalation, leading to the downregulation of Bcl-2 and secretion of caspases, triggering senolysis [73] (Figure 1). The compound induces S-phase cell cycle arrest and subsequent apoptosis in cancer cells, with an associated downregulation of Bcl-xL and Bcl-2, and an upregulation of Bax and cytochrome C [73]. It has induced effective senolysis in other disease models when used in combination with dasatinib, which stimulates proapoptotic caspases through Akt inhibition [74] (Figure 1). Combining quercetin with dasatinib reduced senescent burden in preadipocytes and human umbilical vein endothelial cells (HUVECs) [18]. Additionally, it improved many age-related symptoms in an Ercc1^−/Δ^ mouse model of premature aging, including cardiovascular function, bone density, and physical endurance [18] (Table 1). In the CNS, quercetin reduced neurodegenerative burden in a triple-transgenic mouse model of AD by decreasing amyloid-β expression and alleviating some of the pathology associated with microgliosis and astrogliosis [75]. The dual combination reduced SA-β-Gal expression, several cytokine secretions, the number of reactive microglia, and amyloid-β plaque formation in the hippocampus of the APP/PS1 AD mouse model [52] (Table 1). Furthermore, it reduced atrophy within the cortex and reduced neurofibrillary tangle expression in cortical neurons, while improving cerebral blood flow in a transgenic mouse model of AD [51] (Table 1). Finally, combining quercetin and dasatinib administration improved cognitive function and reduced the number of lamin B1-deficient neurons in a mouse model of normal aging [55] (Table 1). Given the beneficial effects of PI3K-targeting senolytics in these disorders, and their tolerability in clinical trials for other disorders [76,77], trialling the impact of these drugs on glial senolysis in ALS may be a useful strategy.

However, one potential caveat is that upregulating PI3K activity in SOD1^G93A^ NSC34 cells [78] and triggering Akt activation in cultured motor neurons from the spinal cords of SOD1^G93A^ mice [79] improved cell viability, suggesting that inhibiting PI3K activity may have unwanted effects on neurons in the disease (Figure 2). Similarly, activation of the PI3K/Akt pathway in SOD1^G93A^ mice resulted in improved motor function and a longer lifespan [79,80]. Therefore, any work examining the impact of PI3K-targeting senolytics in ALS should gauge the impact on both senolytic glia and neurons. Furthermore, if quercetin is to be trialled in ALS models, it will need to be shown that intercalation into DNA by quercetin only occurs in senescent cells and not healthy cells. Additionally, quercetin also holds anti-inflammatory properties as it can attenuate microglial activation and reduce levels of TNF-α, IL-1α [81], IL-1β, IL-6, and IL-8 [82]. Thus, it will be crucial to explore whether any potential protective properties of quercetin arise from its senolytic ability or its direct capacity to combat neuroinflammation and oxidative damage [83]. Similarly, if dasatinib is to be trialled in ALS models, off-target effects from its impact on tyrosine kinase receptors will need to be explored [84]. Another factor to consider is that quercetin has uncertain CNS bioavailability. Some studies suggest it has low potential to cross the blood brain barrier (BBB) [85], however, quercetin readily crosses in vitro models of the BBB. If further research confirms that the in vivo BBB-permeability of quercetin is prohibitive, administration through nanoparticles could offer an alternative avenue for effective delivery [85]. In contrast, dasatinib has shown great efficacy in crossing the BBB in cancer treatment within the CNS [86]. Despite differences in bioavailability between the drugs, their combination is currently being trialled in an intervention-based Phase 2 clinical trial in patients with AD [87], which may offer some insight into their safety and efficacy for neurodegenerative disorders in humans.

Inhibition of the anti-apoptotic Bcl-2 family members is a further strategy for senolytic development, with navitoclax developed for this purpose [62] (Figure 1). It successfully induced apoptosis in senescent HUVECs and lung fibroblast (IMR90) cells in vitro [88]. Additionally, it reduced p16 and p21 expression in dorsal root ganglia cells cultured from a cisplatin-induced model of peripheral neuropathy in vitro and improved neuronal functional response to pain in vivo [54] (Table 1). Furthermore, navitoclax treatment showed efficacy in reducing the senescent burden and the associated level of phosphorylated tau protein aggregates in a PS19 mouse model of AD [50] (Table 1). However, due to the role of the Bcl-2 family in preventing cellular apoptosis, the non-selective targeting of the protein members by navitoclax can lead to more widespread cellular death [90] and has resulted in several adverse effecra in humans, including depletion in neutrophil, leukocyte, and platelet populations [91]. The senolytics A1331852 and A1155463 showed the same effects in HUVECs and IMR90 cells as navitoclax (Table 1) but hold the advantage of fewer potential side effects as they are more selective and only target Bcl-xL [89]. Bcl-xL is significantly elevated in senescent cells and thus selectively targeting this Bcl-2 family member is a promising strategy [92]. In addition to its senolytic effects on glia, inhibiting Bcl-2 family members may have further beneficial effects for motor neurons in ALS (Figure 2). Through interactions with SOD1, Bcl-2 can damage the mitochondria and members of the Bcl-2 family mediate motor neuron loss [22,93]. Therefore, inhibiting Bcl-2 family members may be a promising strategy for inducing senolysis in ALS, as well as having a directly beneficial effect on motor neurons. A1331852 and A1155463 have not yet been tested in a CNS-relevant model, so it will be important to screen them in preclinical in vitro and in vivo models of disorders such as ALS to establish efficacy. An alternate future strategy may also be to investigate methods of senescence prevention, although this would require further research as current understanding regarding the exact cause of senescence is limited.

## 5. Conclusions

Glial senescence is displayed in pre-clinical ALS models and post-mortem human ALS tissue. Selective elimination of senescent cells has demonstrated pre-clinical success in other neurodegenerative and ageing-related diseases such as AD, but it has not yet been evaluated in ALS. However, current senolytic strategies such as Hsp90 and PI3K inhibition may counterproductively endanger degenerating motor neurons. In contrast, inhibiting Bcl-2 family members may be able to protect motor neurons both directly, and indirectly, through glial senolysis. In addition, identification of further targets that are selectively upregulated in senescent glia in ALS that could be targeted without impacting motor neurons need to be identified. If such targets can be found, they may one day allow for slowing of ALS progression at much earlier stages of the disease, possibly even extending the lifespan of patients living with this condition.

## Figures and Tables

**Table 1:** A selection of current senolytic agents. M: mouse; V: in vitro; Ercc1−/Δ: ERCC Excision Repair 1-deficient; AD: Alzheimer′s Disease; NFTs: neurofibrillary tangles; HUVECs: human umbilical vein endothelial cells; IMR90: human lung fibroblast cells; PN: peripheral neuropathy; DRG: dorsal root ganglia; ↑ denotes increase/improvement; ↓ denotes decrease.

Senolytic Agent	Mechanism of Action	Effects in Models of non-CNS Disorders	Effects in Models of CNS Disorders
17-DMAG	Hsp90 inhibition	Premature Aging Ercc1^−/Δ^ model [62] ↓ p16-expressing embryonic mouse fibroblasts (V) ↓ p16 and SA-β-Gal (M) ↓ kyphosis; ↑ motor function and coordination (M)	-----
Quercetin (Q)	PI3K inhibition DNA intercalation	Premature Aging Ercc1^−/Δ^ model (M) [18] ↓ p16 and SA-β-Gal ↑ bone density; ↑ ability to exercise	APP/PS1 model of AD (M) [52] ↓ SA-β-Gal; ↓ IL-6; ↓ microglial activation ↓ hippocampal Aβ plaque burden, TNF-α, and IL-1β, ↓ cognitive loss AD (M) [51] ↓ NFTs; ↓ atrophy in cortex ↑ blood flow in cerebellum Normal Aging (M) [55] ↑ maze performance; ↑ lamin B1 in neurons
Dasatinib (D)	Akt inhibition		
Navitoclax	Non-selective Bcl-2 family inhibition	Senescence induced through radiation, oxidative stress, and excessive replication (V) [88] ↑ apoptosis of Bcl-xL- and Bcl-2-expressing HUVECs and IMR90 cells PN induced through cisplatin [54] ↓ p16 and p21 in DRG (V) ↓ mechanical and thermal thresholds of pain (M)	Tau-prone PS19 model of AD (M) [50] ↓ *p16*, *p21*, *IL-6*, and *IL-1β* expression ↓ phosphorylated tau
A1331852 A1155463	Selective Bcl-xL inhibition	Radiation-induced senescence (V) [89] ↑ apoptosis of Bcl-xL-expressing senescent HUVECs and IMR90 cells	-----
